# Design and Application of Portable Centrifuge Inspired by a Hand-Powered Spinning Top

**DOI:** 10.3390/mi14101968

**Published:** 2023-10-22

**Authors:** Dongbao Tang, Ziwei Duan, Luxuan Liu, Zhaoyuan Jia, Lijun Lang, Yuyu Tan

**Affiliations:** 1 School of Electrical Engineering, Ultra-Fast/Micro-Nano Technology and Advanced Laser Manufacturing Key Laboratory of Hunan Province, University of South China, Hengyang 421001, China; 15673341407@163.com (D.T.); liuluxuanllx@163.com (L.L.); zhaoyuvanjia2022@163.com (Z.J.); 2 School of Biomedical Engineering, Shenzhen Campus of Sun Yat-Sen University, Shenzhen 518107, China; duanzw@mail2.sysu.edu.cn; 3 Department of Computer Science and Engineering, Ho Sin-Hang Engineering Building, Chinese University of Hong Kong, Hong Kong 999077, Hong Kong SAR, China; lijunlangxmu@outlook.com

**Keywords:** electricity-free, top-inspired, hand-powered, portable centrifuge

## Abstract

Traditional centrifuges, extensively employed in biology, chemistry, medicine, and other domains for tasks such as blood separation and pathogen extraction, have certain limitations. Their high cost, substantial size, and reliance on electricity restrict their range of application. Contemporary centrifuges, inspired by everyday items like paper trays and egg beaters, boast characteristics such as ease of operation, independence from electricity, and portability. These features offer unique advantages in specific situations, such as electricity shortages, inadequate infrastructure, and challenging medical conditions. Consequently, we designed a hand-powered portable centrifuge driven by pulling a rope. Our experiments revealed significant performance factors, including load capacity, rope length, and frequency of rope pulling. The results demonstrated that the revolutions per minute (RPM) of a hand-powered portable centrifuge were directly proportional to the length of the rope and the frequency of pulling, up to a certain limit, while inversely proportional to the load. When used for separating and washing polystyrene microspheres, the portable centrifuge’s performance equaled that of traditional centrifuges. According to relevant calculations, this centrifuge could be capable of meeting the application of blood separation. Therefore, we believe this portable centrifuge will find meaningful applications in similar areas, particularly in resource-poor settings.

## 1. Introduction

Centrifuges are critical equipment in medical diagnostics, employed for separating plasma from blood, extracting pathogens and parasites from biological fluids such as urine and feces, and for use in immunoassay, blood analysis, and concentration determination experiments [1,2,3]. Centrifugation enables the separation and discarding of unnecessary substances, reducing interference and thereby enhancing the accuracy and reliability of rapid diagnoses crucial in detecting maladies like malaria, HIV, and tuberculosis [4,5,6,7]. Traditional centrifuges, commonly used and driven by motors to achieve high speeds and generate centrifugal force, are capable of layering samples based on density, size, and weight. However, due to their high cost, substantial size, and dependence on electricity, their use is limited under certain conditions. Hence, there is an urgent need to develop portable, electricity-free, and cost-effective centrifuges.

Several researchers have developed hand-powered centrifuges that can reach speeds of up to 600 rpm, constructed using salad spinners for determining hematocrit values in resource-scarce conditions [8]. The sample holder was made of plastic, with the microcapillary firmly fixed at an appropriate position using adhesive tape. However, the speed of this centrifuge was low, and its volume was relatively large [9]. Another study demonstrated how hand-powered centrifuges could be modified from egg beaters to separate plasma from whole human blood for diagnosing infectious diseases. The centrifuge tube was placed on the egg beater paddle, reaching a revolution speed of up to 1200 rpm. This hand-powered centrifuge was suitable for developing countries with scarce resources [10], but the weight of the centrifuge was heavy, and the revolution speed needed improvement. Furthermore, relevant scholars proposed a low-cost paper centrifuge (paperfuge) inspired by an ancient rotary toy. The paperfuge, operated without electricity, consisted of thin paper, a rope, and two wooden handles. The centrifuge tube was affixed to the paper plate, and high-speed rotation was achieved by repeatedly pulling both handles [2]. Additionally, a scholar presented a centrifuge that merely required gentle stirring of a fidget spinner with fingers [11]. The centrifuge tube containing the blood sample was inserted into a symmetrical position in a hollow sleeve, and the fidget spinner was stirred for centrifugal separation. According to experimental analysis, the separation purity of the blood sample post-centrifugation was as high as 99% in less than 8 min. However, the revolution speed still needed to be increased. In recent years, researchers have demonstrated a miniaturized and low-cost centrifuge consisting of a computer hard disk drive with a 12 V rechargeable lithium-ion battery inside, also allowing direct connection to a car battery for operation in a low-infrastructure environment [12]. Interestingly, in order to overcome limited resource conditions, relevant scholars have proposed a low-cost, portable, stable, and safe 3D printed centrifuge (Mobilefuge), powered by a USB port connected to a mobile phone and a power supply [13]. However, power still limits the applicability of these centrifuges in extreme environments.

Despite advancements in manual portable centrifuges, there remains a gap for hand-powered centrifuges that are portable, practical, low-cost, and comparable in performance to conventional centrifuges. Blood, in particular, is usually separated by applying a relative centrifugal force (RCF) of approximately 800 g; the liquid part separated from red blood cells, white blood cells, and platelets contain essential biological molecules like cortisol and fibrin [14,15], providing the raw materials for function tests. Accordingly, we designed a portable centrifuge inspired by a hand-pulled spinning top and driven by a pullcord. This centrifuge has the advantage of operating without electricity while meeting the RCF necessary for serum separation.

## 2. Materials and Methods

### 2.1. Measurement of Portable Centrifuge Speed

In this study, a laser velocimeter equipped with a laser sensor was provided by Suzhou TASI Electronics Co., Ltd.(Suzhou, China) was used to measure the speed of the portable centrifuge. The velocimeter was set to non-contact speed measurement mode for 2 s, and the speed was measured by aiming at the reflective paper attached to the centrifuge. The revolution speed was displayed on the LCD screen after pressing the button.

### 2.2. Performance Test of Portable Centrifuge

A turntable device, loaded with two centrifuge tubes, was placed on the spinning top with a piece of reflective paper affixed to its surface. Initially, the rope was pulled at a speed of 0.5 s with rope lengths of 16 cm, 24 cm, 32 cm, and 40 cm, incrementally increasing the number of rope pulls from 1 to 7 times. The speed was then read using the laser tachometer. This procedure was repeated after replacing the load on the turntable with four and six centrifuge tubes.

### 2.3. Solid–Liquid Separation Experiment of Polystyrene Microsphere Solution

Polystyrene microspheres mixed with PBS (0.01 mM) solution and three 210 μL samples of polystyrene microsphere solution were left to stand. A 10 μL sample of each solution was then observed under a microscope. The remaining 200 μL of each polystyrene microsphere solution was used for solid–liquid separation using a conventional microcentrifuge, the portable centrifuge developed for this study, and a control group. After 5 min of operation, the distribution of polystyrene microspheres in a 10 μL supernatant was observed under a microscope.

Without centrifugation, the solution exhibited a state of turbidity and uniform distribution after the polystyrene microspheres were mixed with the PBS solution, indicating that the polystyrene microspheres were not soluble in the PBS solution. Under the external forces of centrifugation and gravity, the polystyrene microspheres settled at the bottom, leaving the supernatant clear and free of microspheres.

### 2.4. Centrifuge Washing Experiments

Two 300 μL samples of polystyrene microsphere solution were centrifuged using the portable centrifuge for 5 min. The supernatant was subsequently removed in the following quantities: once at 200 μL, twice at 20 μL, four times at 10 μL, and three times at 2 μL, to yield two polystyrene microsphere precipitates. Subsequently, 20 μL of red dye was added and the polystyrene microspheres were mixed with the dye using a vortex mixer and left for 1 h. One part of the mixture was then mixed well with 200 μL of a 10% ethanol solution and 200 μL of water. The mixed solution was operated in the portable centrifuge for 5 min, and the supernatant was finally removed.

## 3. Results and Discussion

### 3.1. Design of Portable Centrifuge

When the spinning top is forced into rotation, it not only turns around its own axis, formed by its symmetrical structure, but also moves around the vertical axis. Due to the effects of air resistance, friction, and gravity, the rotational force gradually weakens, and eventually, the top comes to a halt. In this study, we designed a hand-powered portable centrifuge with a unique turntable structure. The main body of this centrifuge takes inspiration from a hand-launched spinning top, comprised of a magnetic emitter. By repetitively pulling the rope to accumulate velocity, basic centrifugal experiments can be conducted once the revolutions per minute reach a certain value.

**Figure 1:**
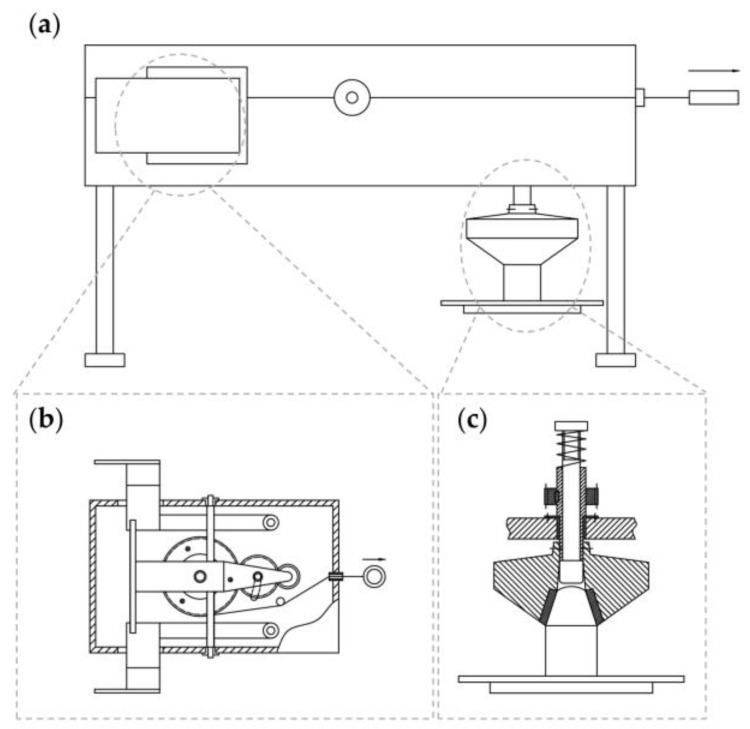
The Schematic Diagram of the Structure Design. (**a**) Overall diagram of the structure and state during rope pulling. (**b**) Structural drawing of internal transmission machinery. (**c**) Diagram of unloading and rotating structure.

The design concept for the turntable part of the portable centrifuge in this study was as follows: First, we considered the structure and size of the horizontal rotary head top [16,17]. Second, to balance the load of the centrifugal samples, we ensured the number of centrifuge tubes with symmetrical structures was even (i.e., two, four, six, and so on). Hence, we first considered the structure and size for loading two centrifuge tubes. Once this was established, similar centrifugal tubes could be added. In summary, as shown in the overall structure in Figure 1a, the disc spring stores energy when the main wheel is pulled by the rope. After the rope is released, the disc spring recovers, driving the main wheel to rotate while the pull rope rewinds back to the winding groove of the active reel (internal transmission is shown in Figure 1b and Figure S1a,b). After completion of the operation, the interior part of the shell is pushed, causing the pusher to rotate around the axis. The turntable and the centrifuge tube are then disassembled, and the centrifuge tube is removed from the insert plate (see Figure 1c and Figure S3a,b,c). We proposed three design schemes for the turntable.

**Figure 2:**
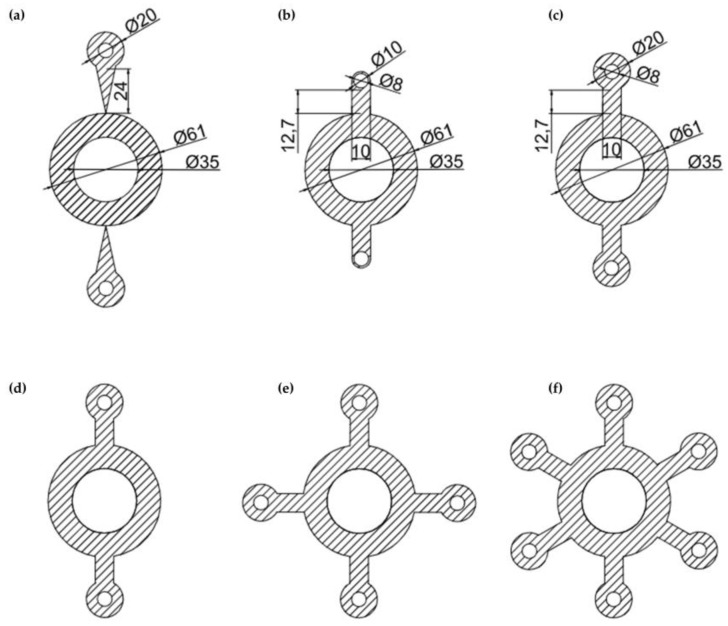
Turntable Design. (**a**) The centrifuge tube loading area and the large ring are connected by a triangle. (**b**) The centrifuge tube loading area and the large ring are connected by a thin frame. (**c**) The centrifuge tube loading area and the large ring are connected by a rectangle with a width of 10 mm. (**d**) The turntable with a loading capacity of 2. (**e**) The turntable with a loading capacity of 4. (**f**) The turntable with a loading capacity of 6.

The first design, depicted in Figure 2a, features a circular position for loading the centrifuge tubes, providing ample support. However, the connecting part with the central large circle is too narrow, and the overall connecting length is too long, making it unstable and prone to breaking. The design in Figure 2b shortens the length of the connecting part to enhance stability. Moreover, drawing upon the sleeve model of the centrifuge, the structures for loading the tubes have been transformed into a hollow circular hole with thin walls, which have been widened vertically. Although the process is quite complex, this change allows the centrifuge tubes to be inserted more securely, preventing them from falling out during centrifugation.

The inner and outer diameters of the loading hole for the centrifuge tube are 8 mm and 20 mm, respectively, with the middle featuring a ring with an inner diameter of 35 mm and an outer diameter of 61 mm. Additionally, the length of the connecting parts has been reduced to 12.7 mm, and the width to 10 mm, which enhances the stability and firmness of the structure and simplifies the overall design (Figure 2c).

In summary, the structure and size for loading the centrifuge tubes have been determined (Figure 2c) and can be extended to a rotary table structure with four or six centrifuge tubes (Figure 2d–f). Ultimately, a portable centrifuge was assembled by affixing a specific rotary table structure to a hand-powered spinning top (refer to Figure S2a). Later measurements of the revolutions per minute showed that the maximum speed for the structure loaded with six centrifuge tubes was approximately 4000 rpm. Consequently, we currently only consider portable centrifuges loaded with two, four, or six centrifuge tubes.

### 3.2. Performance of Portable Centrifuge

**Figure 3:**
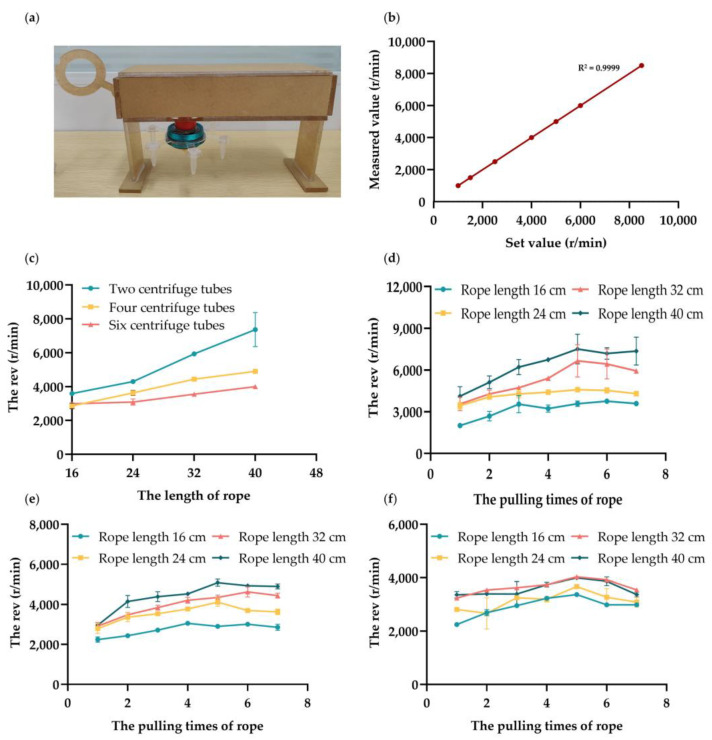
Testing the Revolution Performance of the Hand-Powered Portable Centrifuge. (**a**) The images of the assembled centrifuge. (**b**) Comparison between the measured and set values of the laser velocimeter. (**c**) The effect of the centrifuge tube’s loading capacity and rope length on revolutions per minute. (**d**) The influence of rope length and number of ropes pulls on revolutions per minute when the loading capacity of the centrifuge tube is 2. (**e**) The influence of rope length and number of ropes pulls on revolutions per minute when the loading capacity of the centrifuge tube is 4. (**f**) The influence of rope length and number of ropes pulls on revolutions per minute when the loading capacity of the centrifuge tube is 6.

To verify the reliability of the laser velocimeter, a high-speed refrigerated centrifuge with a wide revolution range was selected as the revolution measuring instrument. The set and measured values of the revolutions per minute were plotted, as shown in Figure 3b. The figure shows a linear trend among sampling points, with a linear regression equation y = 0.9999x − 0.1401, an average error of 3.82, and a regression coefficient of 0.9999. This result indicates that the laser velocimeter can measure revolutions per minute accurately.

To test the performance of the portable centrifuge (Figure 3a), the first step was to identify the relationship between the turntable’s loading capacity, the length of the rope, and the revolutions per minute. The specific operations were as follows: First, we fixed the turntable structures loaded with two, four, and six centrifuge tubes to the spinning top, respectively, placing a centrifuge tube containing the solution on each. We pulled the 16 cm rope five times in 2.5 s, then tested the corresponding revolutions per minute with the laser velocimeter. Second, considering user habits, we changed the rope length to 24 cm, 32 cm, and 40 cm, respectively, and repeated the previous operation. The test data are shown in Figure 3c.

The second step was to study the influence of the number of ropes pulls on the revolutions per minute of the portable centrifuge. When the portable centrifuge was loaded with two, four, and six centrifuge tubes, and matched with rope lengths of 16, 24, 32, and 40 cm, respectively, we varied the number of ropes pulls from one to seven. The corresponding revolution measurement results are shown in Figure 3d–f.

In this study we found that the revolutions per minute of the portable centrifuge with the hand-powered spinning top could meet the needs of a medical low-speed centrifuge and were affected by the following three factors:

1. The greater the number of loaded centrifuge tubes, the lower the revolutions per minute.
2. The longer the rope on the pull-cord, the higher the speed.
3. A rope length of 40 cm and five pulls are the optimal conditions for the three different loads. In addition, the revolutions per minute when loading two centrifuge tubes is the highest.

### 3.3. Application of Portable Centrifuge

We conducted two sets of experiments involving solid–liquid separation and centrifugal washing to demonstrate the effectiveness of the hand-powered portable centrifuge. In the solid–liquid separation experiment, for the control group, we used a microcentrifuge, suitable for various sample filtrations, rapid centrifugal sedimentation, micro blood cell separation, and PCR zonal centrifugation [18,19,20]. The hand-powered portable centrifuge served as the experimental group. We then compared the results of the polystyrene microsphere solution centrifugation experiments from both groups to verify the centrifugation efficiency of the hand-held portable centrifuge.

Polystyrene microspheres are polymerized by free radicals of styrene monomers, and the widely-used polymerization methods are precipitation polymerization, emulsion polymerization, and non-saponifiable polymerization [21,22]. In the study, we selected monodisperse polystyrene microspheres with a diameter of 20 μm to mimic the size of a cell.

**Figure 4:**
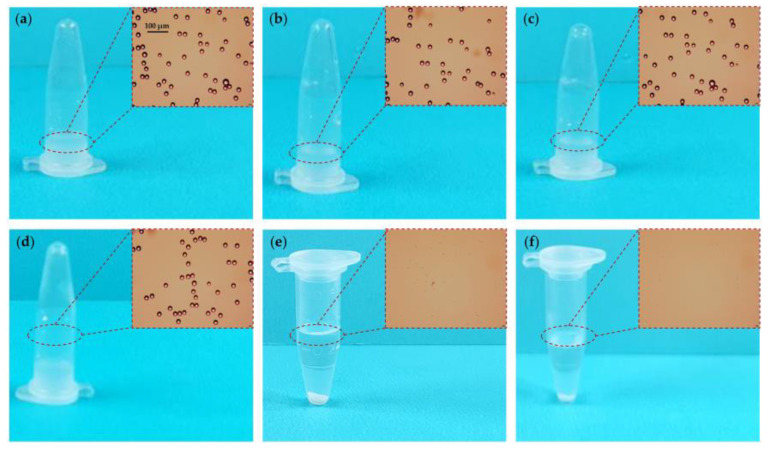
Solid–Liquid Separation Experiment (The inset images show observations under a microscope). (**a**) Polystyrene microsphere solution before centrifugation. (**b**) Polystyrene microsphere solution prior to centrifugation by the microcentrifuge. (**c**) Polystyrene microsphere solution prior to centrifugation by the hand-powered portable centrifuge. (**d**) Polystyrene microsphere solution left standing for 5 min. (**e**) Polystyrene microsphere solution after 5 min of centrifugation by the microcentrifuge. (**f**) Polystyrene microsphere solution after 5 min of centrifugation by the hand-powered portable centrifuge.

Prior to centrifugation, three samples of a monodisperse polystyrene microsphere solution, left standing, appeared relatively turbid in 0.6 mL centrifuge tubes. Under microscopic observation, the polystyrene microspheres were dispersed in the solution (Figure 4a–c). The solution remained turbid after standing for 5 min, with the polystyrene microspheres continuing to disperse in the solution (Figure 4d).

A separation layer could be directly observed in the polystyrene microsphere solution after 5 min of centrifugation by both the microcentrifuge and the hand-powered portable centrifuge. Microscopic observation revealed an absence of polystyrene microspheres in the supernatant (Figure 4e,f). Therefore, the centrifugal effect of the hand-powered portable centrifuge is comparable to that of the microcentrifuge.

The process of centrifugal washing involves placing a solid–liquid mixture into a centrifuge for separation, a method similar to the dewatering mechanism of a washing machine. This leaves the desired solid materials while removing excess liquid. The term ‘washing’ refers to the steps of adding water into the centrifuge tube, dewatering, and then repeating these steps until all impurities are removed [23,24].

In the centrifugal washing experiment, we first separated the polystyrene microspheres from the solution and then dyed them with red dye. Finally, we carried out centrifugal washing experiments using two common washing liquids: ethanol and water in a 0.6 mL centrifuge tube. This allowed us to verify the centrifugal washing effect of the hand-powered portable centrifuge.

**Figure 5:**
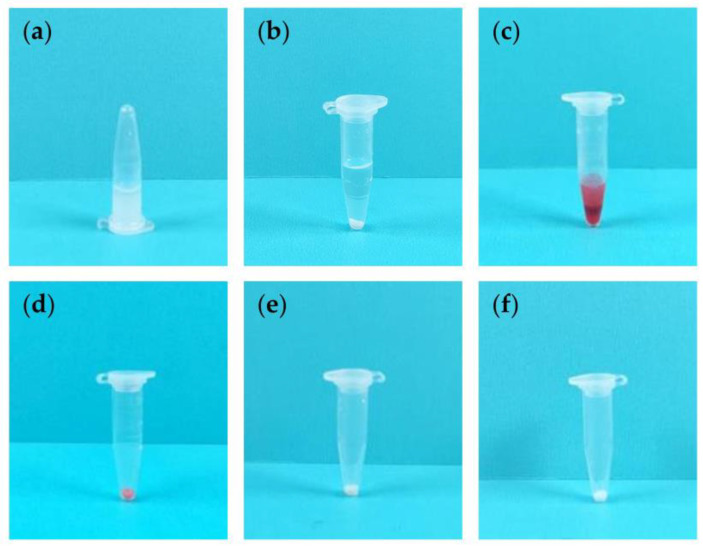
Centrifugal Washing Experiments Using Ethanol. (**a**) Ethanol was used as the washing solution for centrifugal washing. After the non-centrifuged polystyrene microsphere solution was left standing, (**b**,**c**) it was centrifuged by the hand-powered portable centrifuge and subsequently dyed. (**d**–**f**) The dyed polystyrene microsphere solution underwent centrifugal washing with ethanol for the first, second, and third times.

We took a sample of a non-centrifuged polystyrene microsphere solution as the initial test sample (Figure 5a). After centrifugation, we separated the samples into dyed and undyed groups, as shown in Figure 5b,c. We then centrifugally washed the polystyrene microspheres three times using ethanol as the washing solution. By the third wash, the dye had been completely removed by the ethanol, and the color of the polystyrene microspheres returned to its pre-dye state (Figure 5d–f).

**Figure 6:**
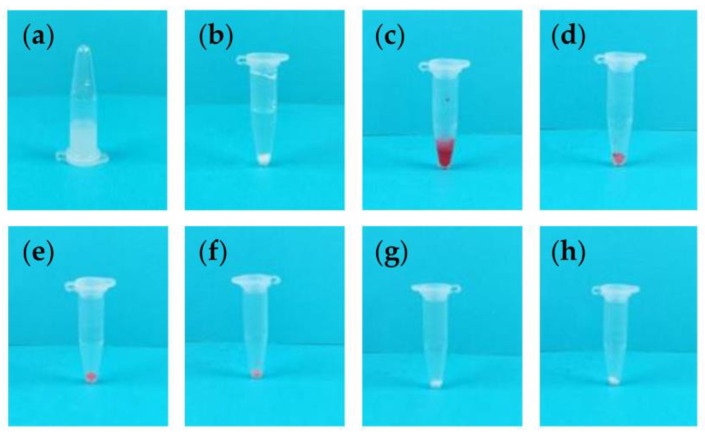
Centrifugal Washing Experiments Using Water. (**a**) Water was used as the washing solution for centrifugal washing. After the non-centrifuged polystyrene microsphere solution was left standing, (**b**,**c**) it was centrifuged by the hand-powered portable centrifuge and subsequently dyed. (**d**–**h**) The dyed polystyrene microsphere solution then underwent five rounds of centrifugal washing with water.

Before centrifugation, the second polystyrene microsphere solution appeared turbid (Figure 6a). After centrifugation, the undyed solution showed a clear separation into layers (Figure 6b). In the dyed solution, a distinct red sediment was observed at the bottom (Figure 6c). Water was used as the washing solution for five rounds of centrifugal washing. The results from the successive washings showed the color of the polystyrene microspheres gradually lightening. By the fifth wash, the dye had been completely removed by the water (Figure 6d–h).

When comparing ethanol and water, ethanol’s centrifugal washing effect was more efficient. Moreover, the dyed polystyrene microspheres could be centrifugally washed to almost the same color as the undyed ones. These results verify that the hand-powered portable centrifuge has the ability to perform basic centrifugal washing.

## 4. Conclusions

This study used a fully manually operated spinning top as the main body of the portable centrifuge, with the addition of a turntable structure for containing samples. The design offers several advantages, including small size, portability, low cost, and durability. We conducted several experiments to test the factors affecting the revolutions per minute of the hand-powered portable centrifuge and compared its application performance. Notably, the hand-powered portable centrifuge was able to effectively complete basic operations such as solid–liquid separation and centrifugal washing, with performance equivalent to that of a traditional microcentrifuge. Moreover, the centrifuge designed in the study was able to reach up to 9000 rpm, corresponding to the RCF 4274 g, which is capable of meeting the application of blood separation. Given its unique features, it shows excellent application prospects in unique environments.

## Data Availability

Not applicable.

## Supplementary Materials

The following supporting information can be downloaded at: https://www.mdpi.com/article/10.3390/mi14101968/s1, Supplementary Materials included description of structure design (Figures S1–S3), operation steps, and editable CAD files (CAD Files S1–S6).
