# Supplementary material for: Design and Application of Portable Centrifuge Inspired by a Hand-Powered Spinning Top
Source: Micromachines (Basel). 2023 Oct 22;14(10):1968. doi: 10.3390/mi14101968 (PMC10609382; doi:10.3390/mi14101968)
Supplement: Supplementary file 1 [file micromachines-14-01968-s001.zip › Supplementary Materials( description of structure design and operation steps).pdf]

Supplementary Materials

Design and Application of Portable Centrifuge Inspired by a Hand-powered Spinning Top

Dongbao Tang [1], Ziwei Duan [2], Luxuan Liu [1], Zhaoyuan Jia [1], Lijun Lang [3] and Yuyu Tan [1,*]

[1] School of Electrical Engineering, Ultra-fast/Micro-nano Technology and Advanced Laser Manufacturing Key Laboratory of Hunan Province, University of South China, Hengyang 421001, China
[2] School of Biomedical Engineering, Shenzhen Campus of Sun Yat-Sen University, Shenzhen 518107, China
[3] Department of Computer Science and Engineering, Ho Sin-Hang Engineering Building, Chinese University of Hong Kong, Shatin, N.T., Hong Kong SAR, Hong Kong 999077, China
* Correspondence: tanyuyu@usc.edu.cn

S1. Description of Structure Design

The power structure consists of several components, including shaft A, the drive wheel structure, disc spring, rope, cannula, output gear, shaft B, and intermediate gear. In terms of structure, shaft A is securely fixed in the inner cavity A of the shaft. The drive wheel structure, which is comprised of a reel and gear, is equipped with a winding groove. This structure can be mounted on shaft A and rotated through the shaft hole. Inside the reel's inner cavity, there is a disc spring that has inner and outer ring end heads at both ends. The inner ring end is connected to shaft A while the outer ring end is connected to the inner cavity B of the reel. The rope is tied to the winding groove of the reel and extends outside the shell along the guide column. The output gear is fixed on the sleeve and the intermediate gear, attached to shaft B, moves along two arc grooves in an arc track motion. It constantly remains engaged with the gear during movement (refer to Figure S1a,b).

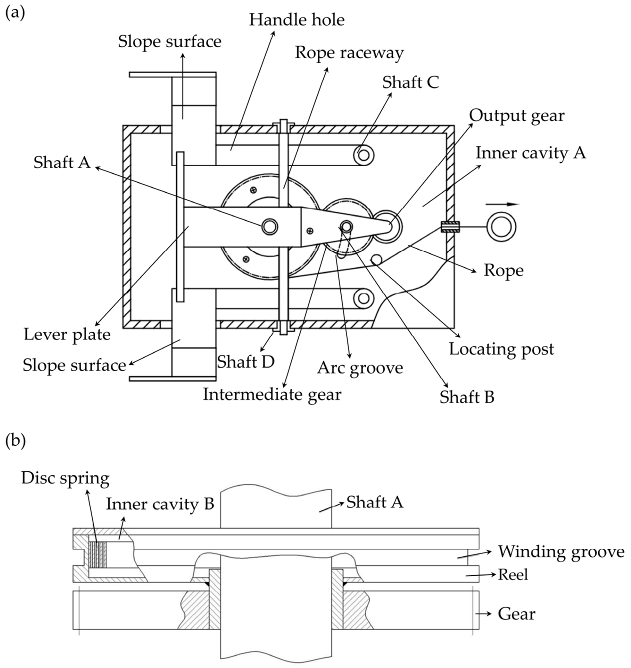

Figure S1 Schematic diagram of the power structure. **(a)** Structure diagram of internal transmission. (**b**) Schematic diagram of installation relationship of shaft A, driving wheel, and disc spring.

The rotating part consists of rotating base, magnet, positioning table, and turntable. The rotating base has an upper and a lower hole. The upper hole is used to insert the rotating base onto the under sleeve, while the lower hole is where multiple magnets are evenly distributed. The positioning table is inserted into the rotating base through the

connecting column and is attracted to the magnets. The turntable, which is made up of a center ring and an outer ring, is connected to the rotating base through the center ring and has centrifugal tubes incorporated into the outer ring (refer to Figure S2a).

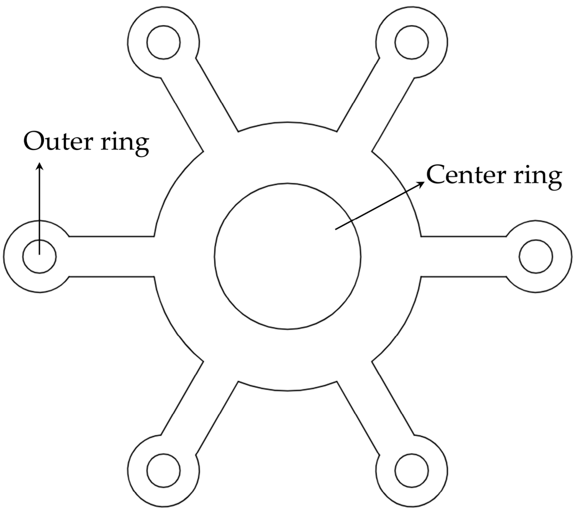

Figure S2. Schematic diagram of the turntable structure.

The unloading part consists of several components, including push switch, shaft C, push rod, nut, spring, warping plate, and shaft D. The push switch is installed in the inner cavity A of the shaft and can rotate at the hinged point through axis C. It also extends along the outside of the shaft. The push rod, which is installed in the cannula, has a threaded top that connects to the push switch. A spring is inserted into the push rod, creating an elastic force that presses the sleeve. When the push switch is triggered, the warping plate engages with the nut. Shaft D is placed inside the inner cavity A of the shaft (refer to Figure S3a–c).

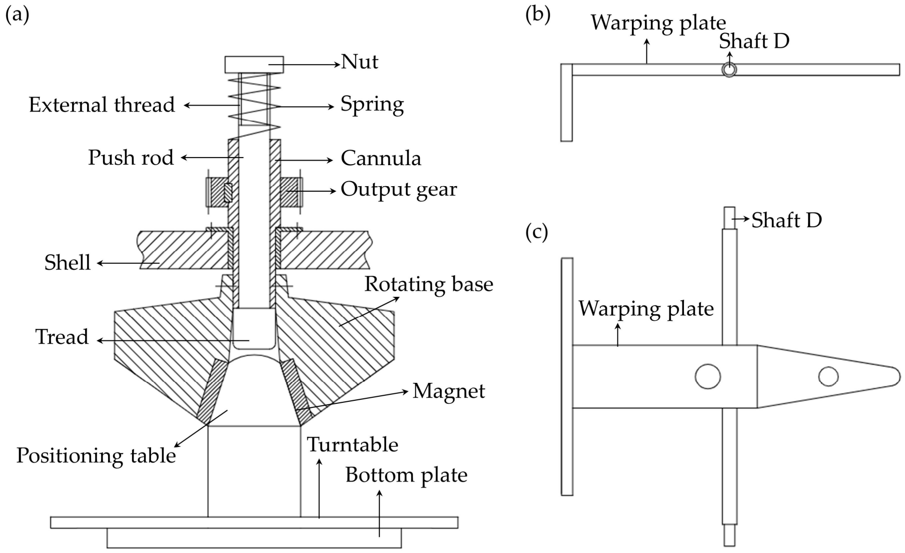

Figure S3. The unloading and the rotating part. **(a)** Schematic diagram of the connection structure between the unloading and the rotating part. **(b)** Schematic diagram of the connection structure between the warping plate and shaft D. **(c)** The top view of Figure S2b.

S2. Operation Steps

Step 1: Prior to centrifugation, the loading amount on the turntable is determined by the number of centrifuge tubes. The centrifuge tubes are then inserted into the outer ring of the turntable.

Step 2: The rope is pulled to drive the reel forward rotation. As a result, the disc spring deforms and stores energy, while the intermediate gear moves to the front end of its arc trajectory. This enables the intermediate gear to engage with both the reel and the output gear simultaneously. The power from the reel is then transferred to the cannula through the intermediate gear and the output gear, causing it to rotate in one direction. When the rope is released, the elastic potential energy in the disc spring is released, causing the reel to reverse rotation until the rope is rewound back into the groove. At the same time, the intermediate gear moves to the back end of its arc trajectory, disconnecting the power transmission between the reel and the intermediate gear.

Step 3: The rotating cannula drives the base, magnet, positioning table and turntable to rotate synchronously, enabling the centrifugal operation of the centrifuge tube.

Step 4: Once the centrifugation process is complete, the push rod is pressed down against the elastic force by activating the push hand switch. This causes the warping plate to remove the positioning table and the turntable with the centrifugal tube.
